# GDM-Related Neurodevelopmental and Neuropsychiatric Disorders in the Mothers and Their Progeny, and the Underlying Mechanisms

**DOI:** 10.3390/jpm16010019

**Published:** 2026-01-04

**Authors:** Zhijin Yan, Jianhong Pu, Dawei Li, Mingxing Liu, Zhice Xu, Jiaqi Tang

**Affiliations:** 1 Institute for Fetology and The Center of Management, The First Affiliated Hospital of Soochow University, Suzhou 215006, China; 20245232065@stu.suda.edu.cn (Z.Y.); pujianhong@suda.edu.cn (J.P.); 2 Reproductive Medicine Center, The First Affiliated Hospital of Soochow University, Suzhou 215006, China; 20214132040@stu.suda.edu.cn; 3 Infection Management Department, The First Affiliated Hospital of Soochow University, Suzhou 215006, China; liumingxing@suda.edu.cn; 4 Wuxi Maternity and Child Health Care Hospital, Wuxi 214002, China

**Keywords:** GDM, neurodevelopment, neuropsychiatric disorders, offspring

## Abstract

Gestational diabetes mellitus (GDM) has witnessed a persistent rise in the prevalence over the past few decades, imposing a substantial burden on global health and economies. GDM exerts both short-term and long-term effects on neuropsychiatric systems of the mothers and their progeny. This review catalogs the neurodevelopmental and neuropsychiatric disorders in GDM women and their offspring and summarizes the possible relationships as well as the underlying mechanisms, which would enhance our understanding of the neuropsychiatric disorders related to GDM, offering information on personalized strategies for patients.

## 1. Introduction

Gestational diabetes mellitus (GDM) is one of the most common complications during pregnancy [1]. The diagnostic criteria vary among guidelines. The criteria given by the World Health Organization (WHO) in 2023 includes fasting plasma glucose ≥ 5.1 mmol/L or 2 h plasma glucose ≥ 8.5 mmol/L after a 75 g oral glucose tolerance test (OGTT) [2]. The criteria given by the International Association of Diabetes and Pregnancy Study Group (IADPSG) includes fasting plasma glucose ≥ 5.1 mmol/L and/or 1 h ≥ 10.0 mmol/L and/or 2 h ≥ 8.5 mmol/L after 75 g OGTT [3].

Neurodevelopment commences as early as the third week of embryogenesis and persists throughout the postnatal period [4]. A growing body of evidence indicated that GDM was a risk factor for neurodevelopmental and neuropsychiatric disorders in mothers and their offspring. However, some studies did not prove the association between GDM and those disorders. This narrative review presented the inconsistent data and discussed the possible influencing factors, such as diagnostic criteria, sample sizes, design of study, GDM treatment, gestational age, birth weight, and so on.

Abnormalities in structures and functions of the brain can give rise to a spectrum of neurodevelopmental and neuropsychiatric disorders [5]. Neurodevelopmental disorders and neuropsychiatric disorders share an overlapping mechanism. Maternal hyperglycemia plays a crucial role in adverse neurologic health. Disruptions in insulin signaling, oxidative stress, inflammation, and epigenetic modification can lead to abnormal neurodevelopment and increase the susceptibility to neuropsychiatric disorders.

Given the increasing prevalence of GDM and its influences on neuropsychiatric health, it is urgent to systematically assess the association between GDM and neurodevelopmental/neuropsychiatric disorders in the mothers and offspring. This narrative review based on the recent literature would offer new perspectives for the personalized managements of GDM-related neurodevelopmental and neuropsychiatric disorders.

## 2. Methods

This review was based on a comprehensive search of the PubMed and Cochrane Library databases. The used keywords included “GDM”, “neurodevelopment”, “neuropsychiatric disorder”, and “offspring/mother”. Neurodevelopmental disorders comprise a heterogeneous group of behaviorally defined conditions characterized by early abnormalities in cognition, motor, language, and/or social development, intellectual development disorders, autism spectrum disorder (ASD), and attention deficit and hyperactivity disorder (ADHD). Neuropsychiatric disorders comprise anxiety, depression, and palsies. These neurodevelopmental and neuropsychiatric disorders, such as cognition, ASD, ADHD, and depression, were also used as keywords.

Cohort studies, animal studies, review articles, and meta-analysis were selected. The literature search was not restricted by any specific publication period. Multiple diagnostic criteria of GDM were included, such as WHO, IADPSG, International Classification of Diseases (ICD), Finnish Gestational Diabetes Study criteria, and so on. Studies that referred to diabetes without explicitly emphasizing GDM were excluded.

## 3. Results

### 3.1. Neuropsychiatric Disorders in Women with GDM

Pregnancy is associated with significant physical and emotional changes along with an elevated risk of neuropsychiatric disorders [6]. Perinatal neuropsychiatric disorders, highly prevalent worldwide, cause suffering and economic and social problems for women and families [7]. GDM poses a significant threat to women’s neuropsychiatric health. Table 1 provides a comprehensive overview of GDM-associated neuropsychiatric disorders, including depression, anxiety, stress-related disorders, cognitive impairment, and dementia, along with the influencing factors.

Maternal depression: Depression and anxiety are the two most common psychiatric disorders during pregnancy and postpartum [8]. Accumulating evidences indicate that women with GDM, regardless of whether it is current or previous, are likely to develop depression [9,10,11,12,13,14], with elevated depression scores [15] and an increased frequency of depressive episodes [16]. These findings are not limited to humans; similar patterns have been observed in animal models [17].

A retrospective cohort study of 54,000 women in Canada reported that, compared to mothers without GDM, those with GDM had a significantly higher risk of being diagnosed with depression during pregnancy and after the first year postpartum [18]. A higher risk of depression was also seen in Bangladesh pregnant women with GDM compared to non-GDM subjects [19]. However, a few cohort studies based on 1043 women in China or 3347 women in America reported no independent association between GDM and maternal depression across all trimesters of pregnancy as well as in the postpartum period [20,21], after adjusting for demographic, clinical, and pregnancy characteristics. The inconsistent results may originate from sample size, different diagnostic criteria, time of assessment, levels of blood glycemia, and antidiabetic medication.

Maternal anxiety and stress: During pregnancy and in the postpartum period, women with GDM had a higher likelihood of developing anxiety, stress, and somatization than women without GDM [15,22,23]. This association was strong in GDM women treated with insulin, but not in those controlled by diet [24,25]. Women with GDM showed higher anxiety scores at 24–34 weeks’ gestation than the non-GDM group, but this difference diminished at around 36 weeks of gestation and during the postpartum period [25,26]. A large cohort study conducted in Canada found that compared to women without GDM, GDM women did not have an increased risk for new-onset anxiety during pregnancy or postpartum [27].

Maternal cognitive decline and dementia: Previous studies reported that women with current or previous GDM had lower scores in cognitive function [28,29,30,31]. The cognitive function was not statistically different in GDM women treated with insulin or managed by only diet [28]. Women with a history of GDM showed a 67% increased risk of incident dementia (hazard ratio 1.67, 95% confidence interval: 1.03–2.69) compared with the control [32]. However, a significant causal relationship between GDM and dementia was not found in the two-sample multivariable Mendelian randomization analysis [33]. The association between GDM and cognitive decline or dementia is complex and many more studies are needed to clarify the relationships.

Influencing factors: These inconsistent findings might be explained by heterogeneity in sample sizes, diagnostic criteria, outcome assessments, time of assessment, physical activity, and GDM management (insulin treatment or diet management).

Women with GDM in the first trimester had a higher risk of depression and anxiety compared to those with late GDM during 24–28 weeks’ gestation and women without GDM [34]. The prevalence of depression in the second trimester was higher in women with GDM (23.4%) than those without GDM (10.7%), but it remained stable from the second trimester to third trimester in both groups [35]. Blood glucose control was another critical determinant. GDM women with uncontrolled blood glucose levels showed higher levels of anxiety, depression, stress, and somatization than GDM women with controlled blood glucose [22]. In stratified analyses based on pre-pregnancy body mass index, significant associations between GDM and depression during pregnancy and postpartum were found only in women from the normal weight group, while no such association was observed in the overweight/obese group [36]. These findings highlighted the importance of considering multiple interacting factors when studying neuropsychiatric disorders in women with GDM, providing valuable insights for the development of targeted intervention strategies.

**Table 1:** Neurodevelopmental/neuropsychiatric disorders in GDM mothers and the influencing factors.

Study Design	Sample Size (GDM vs. non-GDM)	Diagnostic Criteria	Main Effects Odd Ratio (95% Confidence Interval)	Outcome Assessment	Timing of Outcome Assessment	Influencing Factors	References
A prospective cohort study	229 vs. 1220	WHO criteria	Depression scores ↑ at both 1-month and 3-month postpartum	Edinburgh Postnatal Depression Scale (EPDS)	1-month and 3-month postpartum	Higher glucose levels during pregnancy	[10]
A pilot study	382 vs. 366	WHO criteria	Rate of depression ↑ during pregnancy	Montogomery and Asberg Depression Rating Scale	During pregnancy		[19]
A prospective cohort study	105 vs. 108	International Association of Diabetes and Pregnancy Study Group criteria (IADPSG)	Depression scores and rate of developing depression ↑ during pregnancy; No association at 2 and 4 weeks after delivery	EPDS	After the second trimester At 2 and 4 weeks after delivery		[14]
A longitudinal observational study	795 (early 474 + late 321) vs. 1346	IADPSG	Prevalence of depression/anxiety ↑ in early GDM than late GDM and control; Early GDM was significantly associated with depression 1.84 (1.37–2.47) and anxiety 1.36 (1.03–1.79)	Patient Health Questionnaire-9 (PHQ-9)	Early GDM was detected in the first trimester, and late GDM during 24–28 gestational weeks (GW)	Diagnostic time	[34]
A longitudinal study	77 vs. 103	IADPSG	The incidence of depression symptoms in the 2nd trimester ↑; The incidence of depression and anxiety symptomatology -- from 2nd to 3rd trimester	Beck’s Depression Inventory (BDI) State-Trait Anxiety Inventory (STAI)	During pregnancy	Glycemic control Gestational weeks	[35]
A prospective longitudinal observational pilot study	35 (15 insulin treatment + 20 diet management) vs. 20	IADPSG	Depression score -- among three groups; Anxiety score ↑ in GDM-insulin group vs. control; Stress -- between GDM-insulin and GDM-diet groups	Edinburgh Depression Scale (EDS); STAI	During 24–34 GW At > 36 GW	GDM management (insulin vs. diet)	[25]
A population-based cohort study	12,140 vs. 314,583	International Classification of Diseases, version 9/10 (ICD-9, ICD-10)	Prevalence of depression -- Prevalence of anxiety --		During pregnancy and the first year postpartum		[27]
A cross-sectional analysis	425 vs. 1747	ICD-9	Depression scores --	PHQ-9	Postpartum		[20]
A prospective cohort study	150 vs. 916	Finnish Gestational Diabetes Study	Risk of developing depression ↑ 1.70 (1.00–2.89)	EPDS	During third trimester of pregnancy 8 weeks after delivery	BMI in the first trimester Maternal age at delivery	[12]
A retrospective cohort study	29,200 vs. 29,200	Diabetes Canada Clinical Practice Guidelines	Risk of depression ↑ during pregnancy 1.82 (1.28–2.59); No significant difference in the first year postpartum; An 8% increased risk of depression beyond first year postpartum		During 24 weeks gestation up to delivery; In the first year postpartum; Beyond 1 year postpartum	Time of outcome assessment	[18]
A prospective longitudinal study	50 vs. 50	Australasian Diabetes in Pregnancy Society criteria	Anxiety score ↑ in the third trimester Anxiety score -- before delivery and at 6 weeks postpartum	STAI	At the beginning of the third trimester; Antepartum; 6 weeks postpartum	Time of outcome assessment	[26]
A cross sectional case control study	30 vs. 30	IADPSG	Cognitive functions ↓	Montreal Cognitive Assessment (MOCA) Trail Making Test	During 32–36 GW		[30]
A prospective cohort study	1292 vs. 204,171	ICD-10	Risk of dementia ↑ 1.67 (1.03–2.69)	ICD-10	At 38–73 years of age	Physical activity	[32]
Multivariable Mendelian Randomization		Finnish Gestational Diabetes Study	No significant causal relationship between GDM and maternal Alzheimer’s disease or dementia				[33]

Note: Beck’s depression inventory (BDI), Edinburgh Depression Scale (EDS), Edinburgh Postnatal Depression Scale (EPDS), gestational diabetes mellitus (GDM), gestational weeks (GW), International Association of Diabetes and Pregnancy Study Group criteria (IADPSG), International Classification of Diseases (ICD), Montreal cognitive assessment (MOCA), Patient Health Questionnaire-9 (PHQ-9), State-Trait Anxiety Inventory (STAI), World Health Organization (WHO). ↑: increase; ↓: decrease; --: no change.

### 3.2. Neurodevelopmental and Neuropsychiatric Disorders in GDM Progeny

#### 3.2.1. Abnormal Neurodevelopment in Fetuses and Neonates

A wealth of imaging studies had illuminated significant neurodevelopmental alterations in fetuses and neonates exposed to GDM during pregnancy. MRI texture showed that fetuses from mothers with GDM had a smaller corpus callosum and cerebellar vermis, along with a delayed sulci maturation compared to the controls. Notably, these differences were more significant in fetuses from women requiring insulin therapy than those from women requiring diet management [37]. These abnormalities were closely associated with ASD, ADHD, and intellectual disability. Ultrasonographic measurements presented that the widths of posterior lateral ventricles, cavum septum pellucidi, and cisterna magna were higher in the fetuses exposed to GDM than in control group [38]. Neurosonographic parameters indicated that the corpus callosum length and insular and parieto-occipital fissure depths were remarkably elevated in GDM fetuses [39]. Postprandial brain responses to 75 g OGTT were slower in GDM fetuses than in control fetuses [40]. The ratio of frontal antero-posterior diameter to occipito-frontal diameter was found to be higher in GDM fetuses than that in the control [41]. GDM fetuses had a higher pulsatility index of the left fetal middle cerebral artery than the control group [42]. Additionally, GDM affected the development of the brain microstructure of neonate mice [43]. Collectively, those findings strongly suggested that in utero exposure to GDM led to abnormal neurodevelopment in fetuses and neonates, influencing various neuropsychiatric disorders later in life.

#### 3.2.2. Neurodevelopmental Disorders in Offspring

Neurodevelopment is a continuous process from fetuses to postnatal periods. Infants of GDM mothers had microstructural white matter abnormalities, which was associated with worse cognition [44,45]. In GDM-exposed children, specific structural brain changes have been identified. For example, reduced radial thickness in a small, spatially restricted portion of the left inferior body of the hippocampus [46], and decreased global and regional cortical gray matter volume in the bilateral rostral middle frontal gyrus and superior temporal gyrus [47], have been reported. Functional assessments showed that GDM-exposed children exhibited the reduced cortical excitability and long-term depression-like neuroplasticity when compared with control children [48].

Children with GDM exposure exhibited higher hippocampal functional connectivity to the insula and striatum compared to the unexposed children [49]. Children exposed to GDM diagnosed at ≤26 weeks’ gestation showed increased hypothalamic blood flow (a marker of hypothalamic activation) in response to glucose when compared with unexposed children [50]. Exposure to GDM diagnosed before 26 weeks’ gestation was related to enhanced food cue reactivity in the orbital frontal cortex in children and adolescents [51,52]. Compared with the control group, the offspring born to GDM mothers had a significantly higher prevalence of neurodevelopmental disorders at 1 year of age [53].

Abnormal nervous structures and functions were associated with neurodevelopmental/neuropsychiatric disorders. Most studies found there was an association between GDM and neurodevelopmental/neuropsychiatric disorders in the offspring [54], although a few reported no association (Table 2). This section focused on the intelligence disability, communication domain (especially language), ASD, ADHD, motor skills, and other neurodevelopmental disorders in GDM offspring, with epidemiological studies and animal models.

Intellectual disability (ID): The association between GDM and ID in the offspring was inconsistent. Some studies reported that GDM exposure during pregnancy was associated with greater odds of ID in Swedish children [55], and with marginally lower academic performance in Danish children aged 15–16 years [56] and lower intelligence quotient in New York children [57]. Other research indicated that GDM was linked with a decreased risk of ID in Chinese children aged 7–11 years [58]. However, a meta-analysis showed no significant association between GDM and ID risk in offspring [59]. These discrepancies could be attributed to variations in ages, enrolled populations, GDM severity, and others.

Communication domain: Language disorder is one of the important communication disorders, and is a sign of neurodevelopment impairment [60]. GDM was associated with failing the communication domain in the offspring [61]. The association between GDM and impairment in offspring’s language development was contradictory. Most studies reported that children born to GDM mothers showed lower scores in the language domain using the Bayley test [62,63] and showed a lower verbal intelligence quotient [57,64] than the control children. GDM hindered the expressive language in children into adolescent age [65]. However, no significant differences in language development, including comprehension, expressive language skills, and communications, in children born to GDM were also noted [66,67]. The diverse results might be due to ages, regions, and maternal glycemia levels during pregnancy.

ASD: ASD is characterized by impairments in social interaction and communications, and restricted and repetitive behaviors [68]. Its prevalence continues to rise among children [69]. There were a number of epidemiologic studies on the relationship between GDM and ASD. GDM was associated with an elevated prevalence of ASD in children from China and America [58,70,71,72]. However, the association was not confirmed in studies conducted in Spain and Finland [73,74]. A retrospective longitudinal cohort study reported that exposure to maternal GDM diagnosed at ≤26 weeks’ gestation was associated with an elevated prevalence of ASD in children [68]. Another study showed that, compared to diagnoses made before 26 weeks or after 30 weeks of gestation, a GDM diagnosis between 27 and 30 weeks of gestation was associated with the highest risk of children ASD [55]. For mothers with both GDM and obesity, the risk of having a child with ASD was approximately double compared to controls [70]. Additionally, some found that perinatal mental health was associated with increased autism behaviors among GDM children [75]. The differences may originate from regions of enrolled population, diagnostic time of GDM, and maternal statuses.

ADHD: ADHD is an early-onset neurodevelopmental disorder combining overactivity and impulsivity with the inability to concentrate, resulting in functional impairment in academic, family, and social settings. The relationship between GDM and offspring ADHD was inconsistent. Most studies reported that GDM was associated with an elevated risk of ADHD in children from China [58] and Spain [74], as well as in Caucasian children [76]. A meta-analysis showed that children aged 4–6 and 7–10 years born to GDM mothers had a greater risk of developing ADHD [77]. Only a few studies reported that there was no significant association between GDM and children’s ADHD [78,79].

Children born to obese mothers with GDM showed higher scores in ADHD than those born to GDM mothers with normal weight [80]. Both maternal GDM and low socioeconomic statuses were associated with an approximately two-fold increased risk for ADHD in children, but neither children exposed to maternal GDM alone nor low socioeconomic status alone showed a significantly elevated risk for ADHD [57]. A multinational cohort study with linked mother–child pairs reported a higher risk of ADHD in children who were born to mothers with GDM; however, siblings with discordant exposure to GDM in pregnancy had similar risks of ADHD, suggesting that the association between GDM and ADHD in children might not be causal [81].

Motor skills: Motor skills were assessed in offspring, including gross motor skills and fine motor skills. Compared with children of mothers without GDM, children of GDM mothers had a lower motor development quotient [64,82,83]. Fine motor skills, but not gross motor skills, were significantly delayed up to 4 years-of-age in Japanese infants born to GDM women than the control group [67]. However, GDM was not associated with weaker motor skills in 2-year-old Finnish children [84] or in 7-year-old Norwegian children [66]. In addition, at the age of 1.5/2 years, no significant differences were found in fine or gross motor scales in children born to GDM mothers treated with metformin or insulin [85,86].

These various results highlighted the complexity of the relationship, which might be influenced by factors such as study population characteristics, diagnostic criteria, cesarean delivery, birth weight, gestational age, and time of outcome assessment.

Other neurodevelopmental disorders: The association between GDM and neurodevelopmental disorders in offspring was complicated. Some reported that neurodevelopmental delays, particularly in problem-solving ability, processing emotional prosodies, personal skills, and social skills, were remarkably higher in infants born to women with GDM than the control [67,87]. However, others showed that neurodevelopmental disorders (executive functions, perception, memory, sensorimotor, social skills, and emotional/behavioral problems) in children were similar between GDM and the control groups [57,66]. The cognitive profiles, including indexes of verbal comprehension, full-scale intelligence quotient, performance intelligence quotient, perceptual reasoning, working memory, and processing speed, did not differ significantly in children born to GDM mothers between metformin-treated and insulin-treated groups [88]. Intriguingly, Indian children born to GDM women exhibited higher scores in learning, long-term retrieval/storage, and verbal ability than the control group [89].

In animal offspring models, neurodevelopmental disorders were altered by a GDM-like condition. The offspring of streptozotocin (STZ)-induced GDM animals and the control offspring had similar performance in initial visual discrimination and reversal learning, but the STZ offspring took significantly longer to shift to a new strategy [90]. After exposure to GDM by feeding dams a diet high in sucrose and fatty acids, recognition memory was damaged in young adult rat offspring [91]. In a mouse model of diabetes during pregnancy, intrauterine hyperglycemia impaired memory in both the first filial (F1) generation male offspring and their second filial (F2) generation male offspring [92].

Existing evidence from epidemiological studies and animal models indicated that GDM was more likely to affect structures and functions of the brain in fetuses and neonates, with potential implications for neurodevelopment disorders in offspring.

### 3.3. Neuropsychiatric Disorders in GDM Offspring

Neuropsychiatric disorders in offspring include anxiety, depression, and palsy. The GDM-exposed children were reported with significantly higher anxiety levels compared to the control [93,94]. However, one cohort study did not observe anxiety or depression in GDM children [57]. Long-term depressive neuroplasticity in children born to GDM mothers remained inconsistent [48,95]. GDM was not related with cerebral palsy or epilepsy/infantile spasms in children [58,96]. The variations might be caused by sample size, children age, diagnostic criteria, and postnatal factors.

High-fat diets and a STZ-induced GDM animal model showed that anxiety-depression-like behavior was observed in the offspring [97]. Additionally, maternal hyperglycemia induced by STZ during pregnancy did not cause anxiety-like behavior in rat offspring [90,98]. The information in neuropsychiatric disorders related to GDM in offspring was limited and inconsistent. More studies are needed to further determine the relationship between GDM and neuropsychiatric disorders.

### 3.4. Influencing Factors

Multiple factors, such as races, time of GDM diagnosis, levels of maternal hyperglycemia, antidiabetic medication, maternal obesity, offspring gender, children’s age, and social statuses, showed potential influences on the relationship between GDM and offspring neurodevelopmental and neuropsychiatric disorders (Table 2).

Races: A retrospective cohort study found that compared with other races, non-Hispanic white offspring born to GDM mothers had a higher prevalence of speech/language disorder, the combination of speech/language disorders, developmental coordination disorder, learning disability, intellectual difficulty, ASD, and ADSD [99].

Time of GDM diagnosis: When comparing with diagnosis at ≤26 and >30 weeks’ gestation, GDM diagnosed at 27–30 weeks’ gestation was associated with the highest risk of neurodevelopmental disorders, including ID, ASD, and ADHD in children [55]. Another large, clinical cohort study of singleton children at the ages of 3–17 years also found that exposure to maternal GDM diagnosed at 26 weeks’ gestation or earlier, not after 26 weeks, was associated with risk of ASD [68]. However, some studies reported that ADHD, depression, or anxiety risk in children was not associated with gestational age at GDM diagnosis [100,101].

Gender: A gender-stratified analysis revealed a significantly increased risk of ASD only among male offspring of mothers with GDM [71]. Significant neurodevelopmental delays up to 4 years of age were observed only among boys born to mothers with GDM, with no significant increase in adjusted odds for girls [67]. In early pregnancy, maternal hyperglycemia particularly affected the neurodevelopment only in the male offspring, while in late pregnancy, maternal hyperglycemia was significantly associated with lower neurodevelopment in offspring in both genders [102]. Stratified analyses indicated a relationship between GDM and neurodevelopmental disorders in male children only [75]. These suggested that the male offspring born to GDN mothers were more vulnerable to neurodevelopmental disorders than the female offspring.

Level of maternal hyperglycemia: In GDM, compared to persistently low maternal hyperglycemia, moderate and high maternal hyperglycemia was associated with ADHD [103]. Compared to persistently low maternal hyperglycemia, no association was found between intellectual disability in offspring and moderate to high maternal hyperglycemia in mid pregnancy, but high maternal hyperglycemia in early pregnancy was associated with intellectual disability in children [103]. A Chinese birth cohort study found that compared to healthy glycemia group, late pregnancy and full-term hyperglycemia both significantly increased the risk of overall neurodevelopmental delay, whereas early pregnancy hyperglycemia did not [102]. Both the levels and the duration of maternal blood glucose influenced the associations between GDM and offspring neurodevelopment.

Medications: Compared with the control group, the adjusted hazard ratios for ADHD in children were 1.26 (95% CI: 1.14–1.41) for GDM with anti-diabetes medications, and 0.93 (95% CI: 0.86–1.01) for GDM without medications [101]. GDM requiring anti-diabetes medications during pregnancy was associated with risks of depression or anxiety during childhood and adolescence [100]. Additionally, verbal comprehension, receptive communication, expressive communication, academic functioning, and motor skills in children were similar between insulin-treated and metformin-treated GDM women [85,86,88].

**Table 2:** Neurodevelopmental/neuropsychiatric disorders in GDM children and the influencing factors.

Reference	Country	GDM Diagnostic Criteria	Study Design	Sample Size (GDM vs. non-GDM)	Age of Children	Outcomes Odds Ratio (95% CI)	Influencing Factors	Covariate
[55]	Sweden	Clinical diagnosis	Registry cohort	21,325 vs. 2,326,033	6–29 years	Risk of ID, ASD, and ADHD ↑ the strongest associations with ID during 27–30 GW	Time of diagnosis	child sex, birth year, parental education/income/immigration/psychiatric history, birthplace, maternal age, parity, smoking, PCOS and pre-pregnancy BMI
[99]	USA	Clinical diagnosis	Retrospective cohort	1417 vs. 13,063	1.0–6.3 years	non-Hispanic White: Risk of learning disorder, ASD, ID, and speech/language disorders ↑ other races/ethnicities: Risk of neurodevelopmental disorders --	Races	maternal age, race/ethnicity, socioeconomic status, prepregnancy BMI, smoking during pregnancy, preexisting chronic conditions, mental health status, substance use, polycystic ovarian syndrome, birth year, and offspring sex
[66]	Norway	WHO1999	Prospective cohort	72 vs. 194	7 years	Neurodevelopment disorders (motor skills, executive functions, perception, memory, language, social skills and possible emotional/behavioral problems)--		birthweight, child sex, age at follow-up, and maternal socioeconomic status
[59]	Multinational	Clinical diagnosis	Meta-analysis		1–39 years	Risk of ID --		parental age, SES, smoking, BMI, HDP, birth weight, gestational age, and parental psychiatric disorders
[84]	Spain	Carpenter-Coustan	Prospective cohort	68 vs. 169	0.5 & 1.5 years	Language score ↓ Motor skills and Cognition --	Obesity Dietary management	maternal age, education, employment status, marital status, pre-pregnancy smoking status, primiparity, child’s sex, pre-pregnancy BMI (except when it was the independent variable), gestational weeks at delivery, and intervention groups
[89]	India	Carpenter-Coustan	Prospective cohort	32 vs. 483	9.7 years	Learning/language scores ↑		child’s age, sex, gestation, neonatal weight and head circumference, as well as maternal age, parity, BMI, parents’ socioeconomic status, education level, and rural/urban residence
[56]	Denmark	WHO1999	Registry cohort	4286 vs. 501,045	15–16 years	Academic performance ↓	Birth weight	maternal age, parity, conception mode, hypertensive disorders, delivery mode, smoking, nationality, residence, cohabitation, education, offspring sex, birth weight, gestational age/weight cerebral palsy
[67]	Japan	IADPSG	Prospective cohort	2161 vs. 79,543	0.5–4 years	Male: Neurodevelopmental delays (problem-solving ability, fine motor skills, and personal and social skills)↓ Female: Neurodevelopmental delays --	Gender	child’s sex, maternal primiparity, breastfeeding at 6 months, low birth weight, maternal education level, and maternal smoking during pregnancy
[82]	India	IADPSG	Cross-sectional	52 vs. 52	3.5 months	Motor skills ↓ Mental developmental score ↓	GDM management	maternal age, pre-pregnancy weight, infant weight, length, head circumference, and their Z-scores
[83]	Israel	Clinical diagnosis	Prospective cohort	32 vs. 57	5–12 years	Motor skill ↓ Attention deficits ↑ Cognition --	Antidiabetic medications Maternal glycemia control	age, birth order, socioeconomic status, gestational age, and parental education level
[68]	Sweden	ACOG	Registry cohort	25,035 vs. 290,792	6–29 years	Diagnosed at 26 GW or earlier: Risk of ASD ↑1.63 (1.35–1.97) Diagnosed after 26 GW: Risk of ASD -- 0.98 (0.84–1.15)	Time of diagnosis Gestational age at birth	maternal age, parity, education, household income, race/ethnicity, history of comorbidity, child sex, and—in a subgroup—prepregnancy BMI, gestational weight gain, and smoking during pregnancy.
[70]	USA	Clinical diagnosis	Registry cohort	2544 vs. 36,266	0–8 years	Risk of ASD ↑ GDM only: 1.30 (0.80–2.09) Obesity and GDM: 2.53 (1.72–3.73)	Obesity	maternal age at birth, prepregnancy BMI, maternal race, and year of the child’s birth
[71]	China	self-report	Case-control	67 vs. 554	Mean 4 years	Male: Risk of ASD ↑ 3.67 (1.16–11.65) Female: Risk of ASD --	Gender	child sex, gestational age, mode of delivery, parity, maternal education level, and further included prenatal multivitamin use, folic acid intake in the first three months of pregnancy, and assisted reproduction
[65]	Canada	Clinical diagnosis	Prospective cohort	221 vs. 2612	1.5–7 years	Language score ↓	Education	maternal prepregnancy BMI, hypertensive disorders of pregnancy, age, residence, education, monthly income, parity, smoking history, fetal sex, birth weight, delivery mode, and gestational age
[101]	USA	Carpenter-Coustan	Retrospective cohort	29,534 vs. 295,304	≥4 years (median 4.9)	Requiring medication: Risk of ADHD ↑ 1.26 (1.14–1.41) Not requiring medication: Risk of ADHD -- 0.93 (0.86–1.01)	Antidiabetic medications	maternal age at delivery, parity, education, race/ethnicity, household income, maternal history of ADHD, maternal history of comorbidity (cancer or heart, lung, kidney, liver diseases), birth year, and child sex
[76]	Multinational	Clinical diagnosis	Meta-analysis	515 vs. 984,499	4 years–adolescence	Risk of ADHD ↑1.64 (1.25–5.56)	Birth weight	
[79]	China	IADPSG	Prospective cohort	419 vs. 2841	1.5 & 3 years	Risk of autistic traits ↑ 1.49 (1.11–2.00) ADHD symptoms--		pre-pregnancy BMI, hypertensive disorders during pregnancy, maternal age, place of residence, educational level, average monthly income, parity, smoking history, fetal sex, birth weight, delivery mode, and gestational age at birth
[73]	Finland	Clinical diagnosis	Registry cohort	101,696 vs. 543,347	≤11 years	Risk of ASD and ADHD --		birth year, sex, perinatal problems, number of fetuses, mode of delivery, maternal age, parity, marital status, country of birth, smoking history, maternal psychiatric disorders, and systemic inflammatory diseases
[58]	China	Carpenter-Coustan	Registry cohort	90,200 vs. 777,946	7–12 years	Risk of ASD, ADHD, and development delay ↑ Cerebral palsy and epilepsy --	Birth weight	parental age, birth year, child sex, family income, urbanization level, maternal hypertensive disorders, and preterm delivery.
[75]	USA	Clinical diagnosis	Prospective cohort	216 vs. 2163	Mean 4.1 years	Female: Risk of ASD -- Male: Risk of ASD ↑ 3.26 (1.58–6.41)	Maternal depression	maternal race, ethnicity, age at delivery, pre-pregnancy BMI category, child-assigned sex at birth, gestational age category, and age at CBCL assessment.
[100]	USA	Carpenter-Coustan	Retrospective cohort	42,420 vs. 389,854	5–25 years	Requiring medications: Risk of depression and anxiety ↑ Not requiring medications: Risk of depression and anxiety --	Antidiabetic medications	maternal age at delivery, parity, education level, race/ethnicity, household income, maternal history of psychiatric disorders, pre-pregnancy medical comorbidity, smoking during pregnancy, pre-pregnancy body mass index, birth year, and child sex
[96]	Canada	Clinical diagnosis	Registry cohort	81,325 vs. 1,989,148	0–16 years	Risk of cerebral palsy --		maternal age, parity, socioeconomic characteristics (income, drug benefit receipt, residence), infant’s sex, birth year, pregestational hypertension, gestational hypertensive disorders, start of prenatal care, and congenital malformations

Note: Attention deficit and hyperactivity disorder (ADHD), autism spectrum disorder (ASD), ges-tational diabetes mellitus (GDM), gestational weeks (GW), International Association of Diabetes and Pregnancy Study Group criteria (IADPSG), International Classification of Diseases (ICD), intel-lectual disability (ID). ↑: increase; ↓: decrease; --: no change.

Others: The 3.5-years old children born to obese mothers with GDM had higher scores in ADHD than those born to GDM mothers with normal weight [80]. Of note, significantly higher risk effects for ASD and ID in children were reported in obese mothers with GDM [70,73,104]. Children exposed to both maternal GDM and lower socioeconomic status faced an elevated risk for suboptimal neurocognitive development, poorer language development, and ADHD [57]. The combination of GDM exposure and a postnatal high-fat/high-sucrose diets led to atypical inattentive behavior in rat offspring [91]. Birth weight, gestational age, cesarean delivery, and Apgar scores were related to neurodevelopmental and neuropsychiatric disorders, such as cognition, ADHD, and intelligence [105,106,107,108,109]. GDM offspring were more likely to have obesity, large-for-gestational-age, and low Apgar scores [110,111], while GDM mothers had the increased risk of cesarean delivery. However, more studies are needed to clarify the correlation between these factors and neurodevelopmental/neuropsychiatric disorders in GDM offspring.

### 3.5. Mechanisms

**Figure 1:**
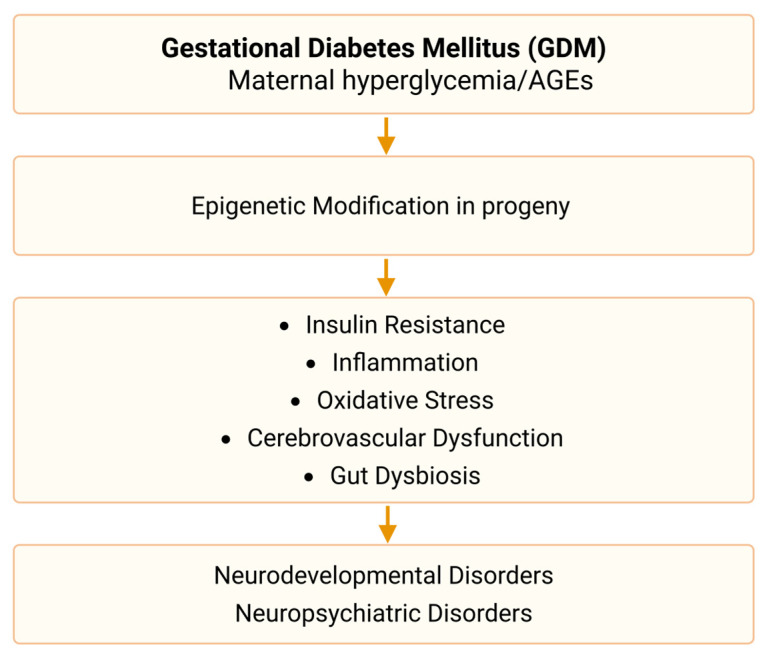
Summary of possible mechanisms in GDM related-neurodevelopmental disorders and neuropsychiatric disorders. Maternal hyperglycemia and/or overproduction of advanced glycation end products (AGEs) could induce insulin resistance [121,122,123,124,125,126], inflammation [90,127,128,129,130], oxida-tive stress [134,135,136], cerebrovascular dysfunction [145,146,147], and gut dysbiosis [148,149,150,151,152,153], finally contributing to neurodevelopmental/neuropsychiatric disorders. Epigenetic modification, especially abnormal DNA methylation, played a significant role in the intergenerational effects of GDM. GDM: gestational diabetes mellitus. AGEs: advanced glycation end products.

Accumulating evidence from human and animal studies have established the association between GDM and neurodevelopmental/neuropsychiatric disorders in both the mothers and their offspring [92,112]. Maternal hyperglycemia, insulin resistance, inflammation, oxidative stress, epigenetic modification, cerebrovascular dysfunction, and gut dysbiosis might be involved in these GDM-related disorders [113] (Figure 1).

Maternal hyperglycemia: Hyperglycemia is an important risk factor for adverse disorders in both mothers and fetuses exposed to GDM. Higher levels of maternal glucose at 1 h and 2 h during OGTT were associated with a higher incidence in neurodevelopmental disorders in children, while fasting glucose levels were not associated with these disorders [114]. Animal studies demonstrated that maternal hyperglycemia induced by STZ during pregnancy induced offspring neurodevelopmental delay, changed hippocampal excitability, impaired memory, disrupted dendritic development, and modified the neurotrophins (crucial for neuronal differentiation, plasticity, and the establishment of synaptogenesis) [92,115,116].

Hyperglycemia conditions during pregnancy disturbed proliferation and cell death of neuroepithelial cells and neural progenitor cells in the developing spinal cord of embryos [117]. Exposure to maternal hyperglycemia also altered the expressions of apoptosis proteins in the hippocampus and triggered neuroinflammation in the forebrain [90,116]. Under hyperglycemic condition, the overproduction of advanced glycation end products (AGEs) might lead to oxidative stress, inflammation, and cerebral blood flow dysfunction, finally negatively affecting neurodevelopment [118,119]. Additionally, hyperglycemia induced persistent oxidative stress, inhibited oxytocin receptor expression, and potentiated maternal diabetes-mediated anxiety-like behavior [120]. These effects were mediated through multiple signaling pathways involving cell proliferation, apoptosis, neuroinflammation, and oxidative stress.

Insulin resistance: Insulin resistance is a key pathological feature of GDM. Insulin resistance is characterized by inactivation of the insulin-signaling pathway, primarily through serine phosphorylation of the insulin receptor substance, thereby disrupting the phosphatidylinositol 3-kinase/protein kinase B (PI3K/AKT) signaling cascade. This disruption blocks glucose transporter 4 translocation and ultimately diminishes glucose uptake. Insulin receptors were expressed in the hippocampus region, a brain region responsible for memory and cognition [121,122]. Abnormal insulin-signaling pathways led to cognition impairment and abnormal hippocampal plasticity via glutamatergic system, hyperphosphorylation of Tau protein, production of amyloid-β (Aβ), and PI3K/AKT [121,123,124]. Maternal insulin resistance before and during treatment was associated with a blunted neuroplastic response in children born to women with GDM [48]. Implantable insulin replacement therapy in the dams effectively reversed the detrimental effects of maternal diabetes on hippocampal excitability, prepulse inhibition, and object-place memory, but not anxiety-like behavior or set-shifting [90]. Metformin therapy could increase insulin sensitivity and improve cognition in a mouse model of GDM [125]. Changes in insulin-like growth factor-1 levels in maternal plasma from GDM pregnancy might affect the neurodevelopment in offspring [126], which might become a potential biomarker of neurodevelopmental disorders in GDM offspring.

Inflammation: A balance between pro- and anti-inflammatory cytokines is crucial in pregnancy. However, GDM was associated with a pro-inflammatory state [127,128]. GDM exacerbated maternal immune activation (such as elevated TNF-α and IL-6). This exacerbated state resulted in a dysregulated transcriptomic profile implicated in inflammatory and neurodevelopmental processes in fetal mouse brain [129,130]. Maternal hyperglycemia during pregnancy decreased IL-1β, increased TNF-α, and the receptor for advanced glycation end-products in the offspring hippocampus [90,116]. Moreover, GDM stimulated microglial activation and neuroinflammation in the offspring’s brain. The effects persisted into young adulthood and contributed to the synaptic and cognitive decline [91]. Neuroinflammation could lead to cognitive impairment, and inflammation had been implicated in neurodevelopmental and neuropsychiatric disorders [131,132,133].

Oxidative stress: The brain is highly vulnerable to oxidative stress due to its high oxygen consumption rate, abundant lipid content, and low anti-oxidant enzymes. GDM was associated with a heightened level of oxidative stress [134,135], which could promote cell damage, disruption of neuronal functions, and loss of synapse, ultimately leading to cognitive dysfunction. Dietary omega 3 polyunsaturated fatty acids (anti-oxidant and anti-inflammatory) had beneficial effects on brain development in GDM-exposed offspring [136]. Reactive oxygen species could negatively regulate insulin-signaling pathway, further contributing to insulin resistance.

Epigenetic modification: Epigenetic modification plays a significant role in the intergenerational effects of GDM. Alterations in both the epigenome and transcriptome have been observed in GDM women and their offspring [137]. Altered DNA methylation in the hippocampus, along with associated genes, contributed to the cognitive impairment in fetuses born to mothers with GDM [138]. Maternal GDM was linked with hypomethylation of ASD-associated gene *OR2L13* promoter in umbilical cord blood [139]. Transcriptomic analysis of F1 and F2 hippocampi indicated a significant enrichment of differentially expressed genes in pathways involved in neurodevelopment and synaptic plasticity [92]. Epigenome-wide association studies revealed that when comparing with non-GDM, there was a differentiated methylation pattern in GDM offspring and mothers [140,141,142,143,144]. These changes in methylation might affect development and alter metabolism. More longitudinal studies are needed to elucidate the underlying complexity of the epigenetic transmission mechanisms between GDM mothers and their offspring.

Cerebrovascular dysfunction: Dysfunction of cerebral blood flow might lead to cognitive impairment. GDM altered fetal middle cerebral artery hemodynamics, including the increase in the ratio of maximum systolic blood flow velocity to end diastolic blood flow velocity, resistance index, and arterial pulsation index, and the decrease in peak systolic velocity at 18–22 weeks of gestation [145,146]. Compared to the control group, fetuses of GDM mothers diagnosed at 26 weeks’ gestation showed an elevated hypothalamic blood flow (a marker of hypothalamic activation) in response to glucose [50]. Hyperglycemia induced abnormal endothelial cells proliferation, thickened capillary walls, decreased perfusion rates, and enhanced vascular permeability [147]. The impaired cerebral hemodynamics, and disturbed structures and functions, may underlie cognitive dysfunction and learning disability in offspring exposed to GDM, highlighting another important mechanism by which GDM influences neurological disorders. Vascular-protective strategies (such as aspirin) may alleviate the neurodevelopmental disorders related to GDM.

Gut dysbiosis: Recent studies supported that GDM affected the maternal microbiome, altered the composition and functions of the gut microbiota, then resulted in gut dysbiosis [148]. GDM could alter the microbiota of the infants during the first year of life [149,150]. The alterations in microbiota of both GDM mothers and neonates shed light on another form of inheritance. The gut dysbiosis modulated the metabolism of fatty acids and amino acids, and further leading to auto-immune diseases and abnormal metabolism [151]. Phytocompound treatment improved gut barrier integrity and reversed dysbiosis in streptozotocin-induced GDM dams, while it also improved cognitive outcomes in GDM offspring [152]. The altered gut microbiome was associated with some mental disorders [153]. More studies are required to further determine the association between neuropsychiatric disorders and gut microbiota in GDM women and progeny.

### 3.6. Intervention

Early identification and management of GDM can improve maternal and offspring health. Despite the implementation of medical treatment during pregnancy, alternative interventions such as dietary modification (nutrients supplement and probiotics), exercise, and good nursing care have emerged as effective strategies with substantial beneficial impacts on the nervous system of both the mothers with GDM and their offspring [154]. Strict glycemic control, healthy lifestyle promotion, and psychosocial support are recommended for GDM women and offspring.

Dietary modification: Dietary modification plays a role in mitigating the adverse effects of GDM. A diet of superior quality, characterized by increased fish consumption, higher maternal intake of food folate, and elevated choline levels during pregnancy, was linked with improved language development [84] and enhanced cognitive scores in children born to GDM mothers [62]. Moreover, the importance of specific nutrients was further highlighted by the discovery that vitamin D deficiency exacerbated autism-related phenotypes in diabetic mice offspring through epigenetic mechanisms [155]. Enhancing genomic vitamin D signaling in human neural progenitor cells protected against hyperglycemia-induced oxidative stress and inflammation [155]. Dietary omega 3 polyunsaturated fatty acids had beneficial effects on brain development of STZ-induced GDM rat offspring [136]. The follow-up study reported that probiotics administration during pregnancy, such as Fusobacteria and Actinobacteria, might contribute to the enhancement of children’s neurodevelopment [80]. However, more studies are expected to explore the relationship between dietary modification and neurodevelopmental/neuropsychiatric disorders related to GDM.

Exercise: Exercise serves as another valuable approach. Physical activity modified the association between GDM exposure and dementia. For mothers with GDM, brisk walking for 30–45 min three times per week might optimize the treatment plan and mitigate anxiety [156]. In the context of offspring, moderate-to-vigorous physical activity during childhood could reduce mental disorders related to in utero GDM exposure [93].

Good nursing: Good nursing is equally essential in managing GDM. High-quality nursing care could regulate blood glucose levels and improve the psychological state of GDM patients [157]. Psychosocial supportive interventions to women with GDM could ameliorate depression, anxiety and stress, also with an influence on enhancing self-efficacy [158]. Additionally, mobile health-based lifestyle interventions showed remarkable effectiveness in improving the mental health of pregnant GDM women [159].

## 4. Discussion and Conclusions

This narrative review elucidated the multifaceted connections between GDM and neurodevelopmental/neuropsychiatric disorders, including depression, anxiety, stress, cognitive impairment, dementia, intelligence disability, communication domain, ASD, ADHD, motor skills, and other neurodevelopmental disorders, and revealed a complex web of influencing factors. Accumulating epidemiological and experimental evidence demonstrated the associations between GDM and those disorders in both the mothers and their offspring. However, reports of no associations also were noted. Such inconsistent results among various studies might be affected by multiple complicated factors, including sample sizes, study designs, diagnostic criteria, time of diagnosis, varying assessments, medication, level of blood glycemia, and obesity. Future work can focus on longitudinal cohorts of the offspring from the young children period to adolescence and adult stages, randomized trials comparing treatment modalities (diet, metformin, and insulin), and multi-omics approaches to elucidate causality.

Several mechanisms underlying the associations of GDM and neuro-disorders included maternal hyperglycemia, insulin resistance, oxidative stress, inflammation, epigenetic modifications, and cerebrovascular dysfunction. This review also highlighted several promising rescue strategies (dietary modification, exercise, and high-quality nursing) that might mitigate the adverse effects of GDM on neuropsychiatric health. The precise molecular mechanisms and personalized management strategies are worth further investigation. Such efforts will be crucial for optimizing the neurodevelopmental/neuropsychiatric disorders in both GDM mothers and their offspring.

In summary, this review linked GDM to neurodevelopmental/neuropsychiatric disorders in both the mothers and their offspring, and discussed the underlying mechanisms, as well as influencing factors. This enhances our understanding of GDM-related neurodevelopmental/neuropsychiatric disorders, and it also offers insights into optimizing GDM screening and developing personalized management strategies during pregnancy and the postpartum period. Early identification and management of GDM could improve maternal and offspring health in personalized medicine for that group of patients. Strict glycemic control, healthy lifestyle promotion, and psychosocial support are recommended for GDM women and their offspring.

## Data Availability

No new data were created or analyzed in this study. Data sharing is not applicable to this article.
